# Rashba coupling amplification by a staggered crystal field

**DOI:** 10.1038/ncomms11258

**Published:** 2016-04-19

**Authors:** David Santos-Cottin, Michele Casula, Gabriel Lantz, Yannick Klein, Luca Petaccia, Patrick Le Fèvre, François Bertran, Evangelos Papalazarou, Marino Marsi, Andrea Gauzzi

**Affiliations:** 1 IMPMC, Sorbonne Universités, Université Pierre et Marie Curie, CNRS, IRD, MNHN, 4 place Jussieu, 75252 Paris, France; 2 Laboratoire de Physique des Solides, CNRS, Université Paris-Sud, Université Paris-Saclay, 91405 Orsay, France; 3 Elettra Sincrotrone Trieste, Strada Statale 14 km 163.5, 34149 Trieste, Italy; 4 Synchrotron SOLEIL, L'Orme des Merisiers, BP 48 Saint-Aubin, 91192 Gif-sur-Yvette Cedex, France

## Abstract

There has been increasing interest in materials where relativistic effects induce non-trivial electronic states with promise for spintronics applications. One example is the splitting of bands with opposite spin chirality produced by the Rashba spin-orbit coupling in asymmetric potentials. Sizable splittings have been hitherto obtained using either heavy elements, where this coupling is intrinsically strong, or large surface electric fields. Here by means of angular resolved photoemission spectroscopy and first-principles calculations, we give evidence of a large Rashba coupling of 0.25 eV Å, leading to a remarkable band splitting up to 0.15 eV with hidden spin-chiral polarization in centrosymmetric BaNiS_2_. This is explained by a huge staggered crystal field of 1.4 V Å^−1^, produced by a gliding plane symmetry, that breaks inversion symmetry at the Ni site. This unexpected result in the absence of heavy elements demonstrates an effective mechanism of Rashba coupling amplification that may foster spin-orbit band engineering.

## Abstract (web summary)

Future spintronic devices may exploit spin-orbit interactions, which often emerge from broken symmetries and strongly influence electronic behaviour. Here, the authors evidence the amplification of Rashba coupling by a crystal field that breaks the local inversion symmetry at the Ni site in BaNiS_2_.

## Graphical abstract

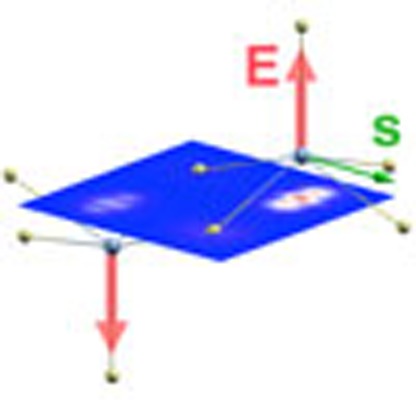

The discovery of the integer quantum Hall effect in field-effect transistors^1^ initiated a novel approach to the electronic properties of crystalline solids based on the topological properties of Bloch states^2^, which fostered the discovery of further remarkable effects, such as the fractional quantum Hall effect^3^, the quantum spin Hall effect^4^ and charge antilocalization^5^,^6^. These effects suggest novel device concepts for quantum computation and spintronics, where information is carried without dissipation by using spin, instead of charge, currents^7^. In this context, there has been an increasing interest in systems where the spin-orbit (SO) coupling leads to the opening of gaps at the Fermi surface and to spin-polarized bands^8^. An attractive possibility is the splitting of electronic states with opposite spin chirality 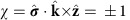 in nonmagnetic materials by the SO Rashba effect. The Rashba Hamiltonian, 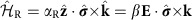, where and *β* are constants and 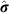 is the spin vector operator composed of the Pauli matrices 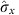, 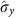 and 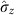, requires an electric field 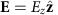 that breaks the inversion symmetry along the *z*-direction perpendicular to the plane containing the spin **σ** and the electron wave vector **k**.

It follows that typical Rashba systems are either bulk noncentrosymmetric crystals or surfaces, referred to as bulk or surface inversion asymmetry systems, respectively^8^. Notable examples are the layered polar semiconductor BiTeI^9^ and the two-dimensional electron gas formed at the surface of SrTiO_3_ (ref. 10), where Rashba splittings as large as 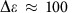 meV have been reported. These large splittings are due to the presence of heavy elements (and thus to large SO couplings) in the former case and to a large electric field *E*_*z*_≈10 mV Å^−1^ at the surface in the latter case. In fact, as noted recently by Zhang *et al*.^11^, SO coupling effects are governed by the local symmetry of the potential felt by the electron, rather than by the symmetry of the bulk crystal. To the best of our knowledge, the first experimental evidence of this circumstance has been given by Riley *et al*.^12^ who observed spin-polarized bands in the layered system 2H-WSe_2_. This polarization, not expected in the centrosymmetric *P*6_3_/mmc structure of 2H-WSe_2_, is explained by the confinement of the electron states within a single WSe_2_ layer with local inversion asymmetry (LIA)^13^.

In the present work, we consider the quasi-two-dimensional semi-metal BaNiS_2_ (ref. 14), precursor of the metal–insulator transition (MIT) observed in the BaCo_1−*x*_Ni_*x*_S_2_ system on the partial substitution of Ni for Co at *x*_cr_=0.22 (ref. 15). This MIT has attracted a great deal of interest for the similarity of its temperature-doping (*T*-*x*) phase diagram to that of cuprate^16^, pnictide^17^ and heavy-fermion^18^ superconductors, though it remains little studied. To the best of our knowledge, the experimental band structure is not available for BaNiS_2_ and first-principles calculations have been hitherto limited to a simple density functional theory (DFT) approach in the local density approximation neglecting the Hubbard repulsion term, *U*, and relativistic SO effects^19^,^20^. The strong local Coulomb repulsion is expected to play a crucial role on the MIT, as indicated by dynamical mean field theory calculations on the insulating phase BaCoS_2_ (refs 21, 22). To elucidate these open issues, the objective of the present study is to provide a first thorough description of the electronic structure of BaNiS_2_. Previous calculations show that one challenge is a complex multiband structure. On the other hand, a favourable condition of this study is the absence of disorder caused by chemical substitutions and a simple tetragonal structure. By means of angular-resolved photoemission spectroscopy (ARPES) on high-quality single crystals, we find a very large Rashba splitting up to Δ*ɛ*≈150 meV, which is unexpected in a centrosymmetric system without electronically active heavy elements. This observation is explained by a very effective LIA mechanism associated with a peculiar non-symmorphic square-pyramidal structure with a gliding plane symmetry. Unlike WSe_2_, in the present case, the large splittings observed are not due to the presence of heavy elements but to a huge staggered crystal field at the Ni site, *E*_*z*_≈1.4 eV Å^−1^, associated with the pyramidal coordination of the Ni ions. The mechanism unveiled by the present results, therefore, opens new possibilities to tune effectively the topological order of solids without the constraint of using either heavy elements or external fields.

## Results

### *Ab initio* calculations

**Figure 1:**
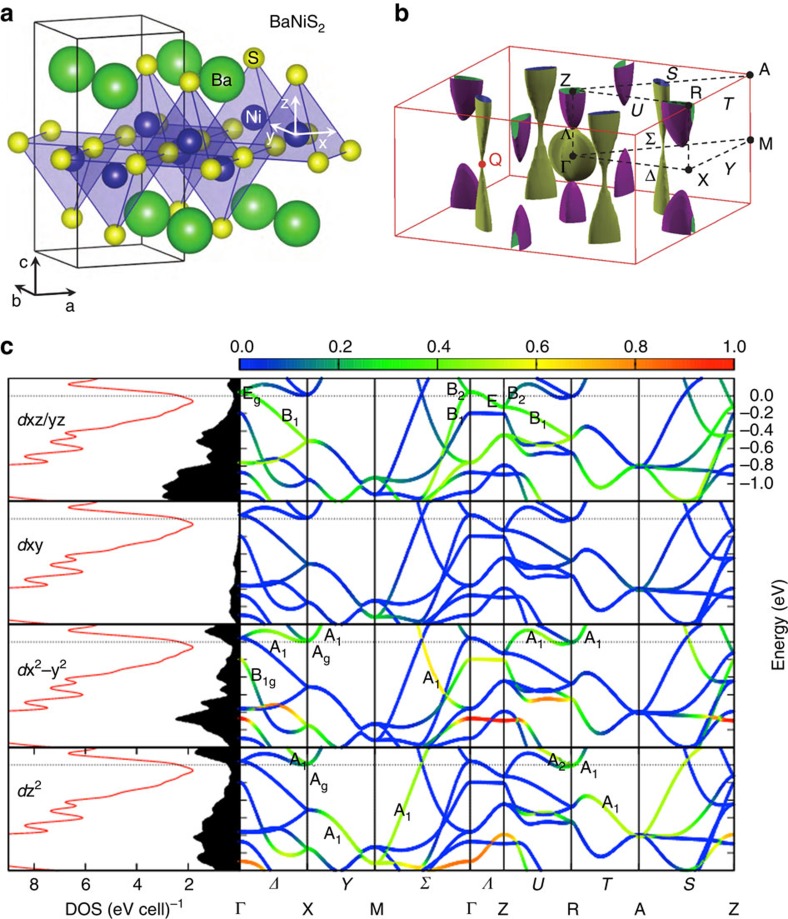
Crystal and electronic structures of BaNiS_2_. (**a**) Tetragonal P4/nmm structure of BaNiS_2_. Note the staggered orientation of the NiS_5_ pyramids. Details of the structure are given in ref. 14. (**b**) Fermi surface calculated in the GGA+*U* approximation without spin-orbit coupling. Hole and electron pockets are indicated in yellow and purple, respectively. (**c**) Calculated electronic bands, total (red line) and projected (black area) density of states for each 3*d* orbital. False colours indicate the *d*-orbital component for each band. The symmetry character of the relevant bands near the Fermi level, indicated by a dotted line, are labelled in Schönflies notation. Bold (italic) letters on the horizontal axis indicate high-symmetry points (lines).

Before presenting the ARPES results and the evidence of Rashba effect, we recall the salient features of the electronic structure of BaNiS_2_ that we recalculated in the generalized gradient approximation (GGA) of DFT supplemented by the Hubbard repulsion, *U*, and by the Hund coupling, *J*. In Fig. 1a–c, we show the crystal structure, the calculated Fermi surface, the band structure and the total and projected density of states (DOS) for each *d*-orbital of Ni. The parameters of the local interaction matrix (*U*=3 eV, *J*=0.95 eV) were estimated from absorption data^23^. In the band structure of Fig. 1c, false colours represent the *d*-orbital component of each band. We use a reference system for the *d*-orbitals where the *x* (*y*) axis coincides with the *a* (*b*) axis, while the *z* axis coincides with the *c* axis and points towards the apical sulphur atom. All *d*-orbitals, except the low-lying *xy* one, contribute to the DOS at the Fermi level, *ɛ*_F_, and hybridize strongly with the 3*p* orbitals of the sulphur atoms. In Fig. 1c, the relevant bands near *ɛ*_F_ are labelled in Schönflies notation for the irreducible representations (IR) of the little point group of the corresponding **k**-vector^24^. We first note two degenerate hole-like bands with E_g_ (*d*_*xz*_ and *d*_*yz*_) character at the D_4h_ symmetry Γ point and with E character along the Λ direction of C_4v_ symmetry; along the Δ (or *U*) and Σ (or *S*) directions of lower C_2v_ symmetry, these bands are split into B_1_ and B_2_ bands. Second, at X (or R) two bands with A_g_ character carrying 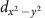 and 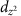 components split into two A_1_ bands along Δ (or *U*). Finally, along Σ (or *S*), two A_1_ electron-like bands carrying 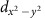 and 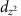 components, respectively, display a linear dispersion and cross at a point denoted Q, located almost exactly at *ɛ*_F_ between the Γ and M points (or between Z and A).

The resulting Fermi surface consists of four sheets. The E_g_ band forms a hole pocket centred at Γ with a peculiar shape similar to that of a carambola fruit. As a result of the *k*_*z*_ dispersion along Λ, this band forms an electron pocket centred at Z with square-shaped section. The A_*g*_ bands form a strongly oblated electron pocket centred at R. Finally, the two linearly dispersing A_1_ bands with 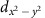 and 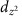 component form a Dirac-like cone with elliptic-like section and hole character. The origin Q of the cone is located near *k*_*z*_=0. All pockets are small, which leads to semimetallic properties, as apparent from the pronounced dip of the DOS at *ɛ*_F_, and to enhanced electronic correlations. Each band is at least twofold degenerate in the orbital sector since the glide reflection plane of the P4/nmm symmetry generates two Ni positions, ***r***_1_=(1/4,1/4,*z*) and ***r***_2_=(3/4,3/4,−*z*), in the unit cell.

### Angular resolved photoemission spectroscopy

**Figure 2:**
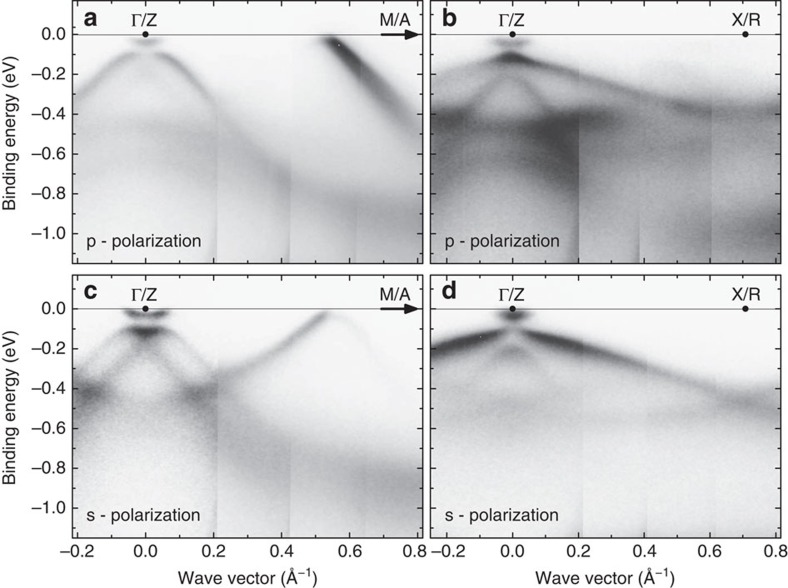
ARPES bands of BaNiS_2_. The band dispersion is shown along the ΓM (ZA) and ΓX (ZR) symmetry directions obtained in both *p* (**a**,**b**) and *s* (**c**,**d**) polarizations using a 26 eV photon energy. Since the *k*_*z*_ value probed is between 0 and π/*c*, symmetry points and symmetry directions referring to both values are indicated.

To obtain high-quality spectra, we optimized the growth of BaNiS_2_ single crystals as described in the experimental methods section. We successfully measured ARPES spectra on several high-purity single crystals cleaved *in situ* at low temperature within the *ab* plane, which allowed us to measure the in-plane band dispersion, *ɛ*_**k**_. As an indication of the improved purity of these crystals, resistivity measurements (see Methods section) yielded residual resistivity ratios *RRR*≈12–17 significantly larger than those ∼4 reported previously^14^,^25^. In Fig. 2, we show the 26 eV ARPES bands measured in both *p*- and *s*-polarizations along the Δ (or *U*) and Σ (or *S*) symmetry directions. Consistent with the orbital character of the bands calculated without SO coupling (Fig. 1c), due to the different parity of the *d*-orbitals with respect to the scattering plane, the 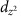 states are observed only in *p*-polarization along Σ, the 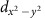 state in *p*-polarization along Δ and in *s*-polarization along Σ and the *d*_*yz*_ and *d*_*xz*_ states in both polarizations along Δ.

**Figure 3:**
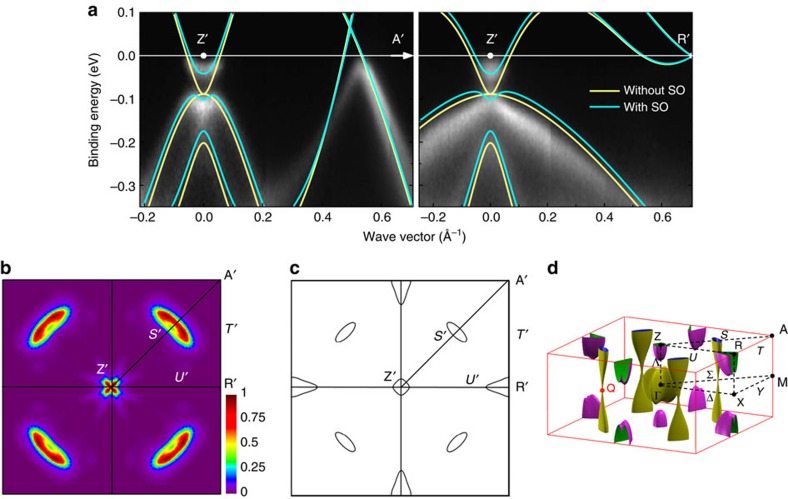
Comparison with calculations. (**a**) 26 eV ARPES bands compared with the GGA+*U* bands along the Z′-A′-R′ directions, corresponding to 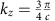, calculated with (light blue line) and without (yellow line) spin-orbit coupling. The ARPES bands are obtained by merging the *s* and *p* polarized spectra of Fig. 2. The horizontal white line indicates *ɛ*_F_. Fermi surface measured experimentally (**b**) and calculated (**c**) at 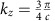. (**d**) Full GGA+*U* Fermi surface calculated with spin-orbit coupling. Hole and electron pockets are indicated in yellow and purple, respectively.

A quantitative comparison between experimental and calculated bands is shown in Fig. 3a, where the previous GGA+*U* bands are plotted together with the full ARPES band structure obtained by merging the *p*- and *s*-polarized spectra of Fig. 2. We noticed that the inclusion of the Hubbard repulsion *U* term in the GGA calculations improves significantly the agreement with the experiment, especially concerning the energy position of the B_1g_ band at Γ (or Z) and the dispersion of the B_1_ band along Γ–X (or Z–R). To estimate the *k*_*z*_ value, we calculated the band dispersion at different *k*_*z*_ values, *k*_*z*_=0, 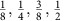 (in crystal units of 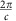), and best agreement with the experiment is obtained for 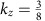. In Fig. 3a–c, the corresponding symmetry points and lines are labelled using primed letters.

**Figure 4:**
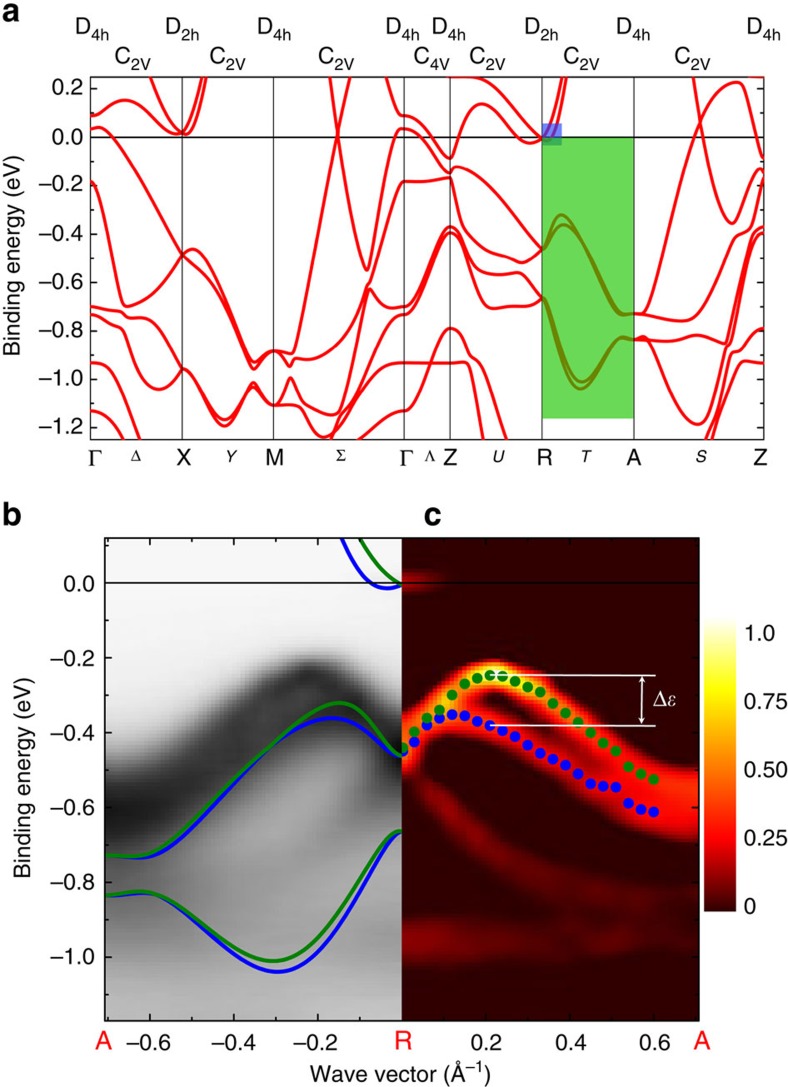
Rashba-split bands. (**a**) GGA+*U* band structure calculated with spin-orbit coupling along the main symmetry directions. The little groups of the symmetry points and lines are indicated on the top. The green and blue areas indicate the portions of the band structure shown in detail in Figs 4b and 5a, respectively. (**b**) Close-up of the calculated bands along the R-A symmetry direction (that is, for *k*_*z*_=π/*c*) plotted together with the experimental ARPES bands measured using 100 eV photons. Blue and green solid lines show the calculated Rashba-split band. (**c**) The experimental splitting of the hole-like band is put into evidence by plotting the second derivative of the energy distribution curves of the spectra of **b** at constant wave vector. Blue and green dots represent the peak position of the second derivative. The energy splitting, Δ*ɛ*≈150 meV, is indicated by white horizontal lines.

As predicted by the calculations for 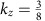, the experimental Fermi surface of Fig. 3b consists of the four hole pockets of the Dirac-like bands along *S*' and of the square-shaped electron pocket centred around Z'. A slight discrepancy between experiment and calculations concerns the hole pocket centred at R', not seen experimentally. According to the calculations, this may suggest that the *k*_*z*_ value probed by the 26 eV photons rather lies in the 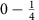 *k*_*z*_ range. In fact, in the calculated Fermi surface of Fig. 3d, both hole pockets at Z and at R shrink rapidly with decreasing *k*_*z*_, so an accurate determination of the relative *k*_*z*_ position of the bottom of these pockets goes beyond the capabilities of the present DFT method. In summary, the experimental data are explained by a pocket at R located at a higher *k*_*z*_ value than calculated. This explanation is supported by the fact that the 100 ev ARPES data do probe the bottom of the pocket, as seen in Fig. 4b. We conclude that the 26 eV data of Figs 2, 3 and the 100 eV data of Fig. 4b probe the bands in the vicinity of 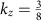 and of 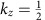, respectively.

## Discussion

We are now in the position of analysing one of the main features of the band structure, that is, the large ≈50 meV splitting of the E_g_ bands at Z, which suggests the importance of SO coupling effects not included in the calculations. A straightforward symmetry analysis^24^,^26^ substantiates this hypothesis. As far as the SO band splittings is concerned, we consider the **k**-points with 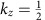, such as Z, on the same footing as the **k′** points with 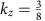, such as Z'. The reason is that the predictions of splitting for the little groups 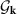 of the former points are valid for the little groups 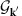 of the latter points as well, as 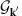 are subgroups of 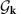. Thus, the symmetry analysis of the SO band splitting is the same for both 26 eV and 100 eV ARPES bands shown in Figs 3a and 4b, respectively. This analysis shows that, while the E_g_ bands are degenerate in the non relativistic calculations, the product of their IR Z_5_ of the D_4h_ little group for the Z symmetry point with the *D*_1/2_ spin IR yields the following sum of IR's:


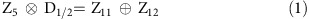


We have then recalculated the band structure including the SO coupling. Remarkably, the result shown in Figs 3a and 4a is fully consistent with the above band splitting. The SO coupling also accounts for the experimental observation of a mixing of the B_1g_ band below the Fermi level with the lower split E_g_ band, clearly visible in the data at Z (Fig. 3a). Indeed, by denoting Z_3_ the IR of the B_1g_ band at Z, one obtains 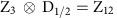, so the two bands are described by the same Z_12_ symmetry in the presence of SO coupling.

The exciting feature of BaNiS_2_ revealed by the present work is that, in addition to the ordinary SO splittings discussed above, both the experimental bands and those calculated with SO coupling display Rashba splittings, as shown in Fig. 4a. We focus on the splitting of the electron-like band with A_1_ character with 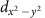 and 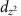 components located just above *ɛ*_F_ near R along *T* (or near X along *Y*) and of the hole-like band located in the same *k* region ≈0.4 eV below *ɛ*_F_. As seen in Fig. 1c, near R the latter band displays B_1_ and B_2_ character with *d*_*xz*_ and *d*_*yz*_ components, respectively, that progressively evolves into A_1_ character with 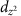 component moving to A. A close-up of the ARPES bands in Fig. 4b shows that only the bottom of the electron-like band is observed, as mentioned above; so the prediction of Rashba effect cannot be verified experimentally for this band but a slight electron doping may be sufficient to probe it. On the other hand, the predicted Rashba splitting is clear in the hole-like band. The splittings in wave vector and in energy are as large as Δ*k*=0.2 Å^−1^ and Δ*ɛ*≈150 meV, respectively. The latter value is even larger than in BiTeI 9 or SrTiO_3_ (ref. 10), in spite of the absence of heavy elements or surfaces.

**Figure 5:**
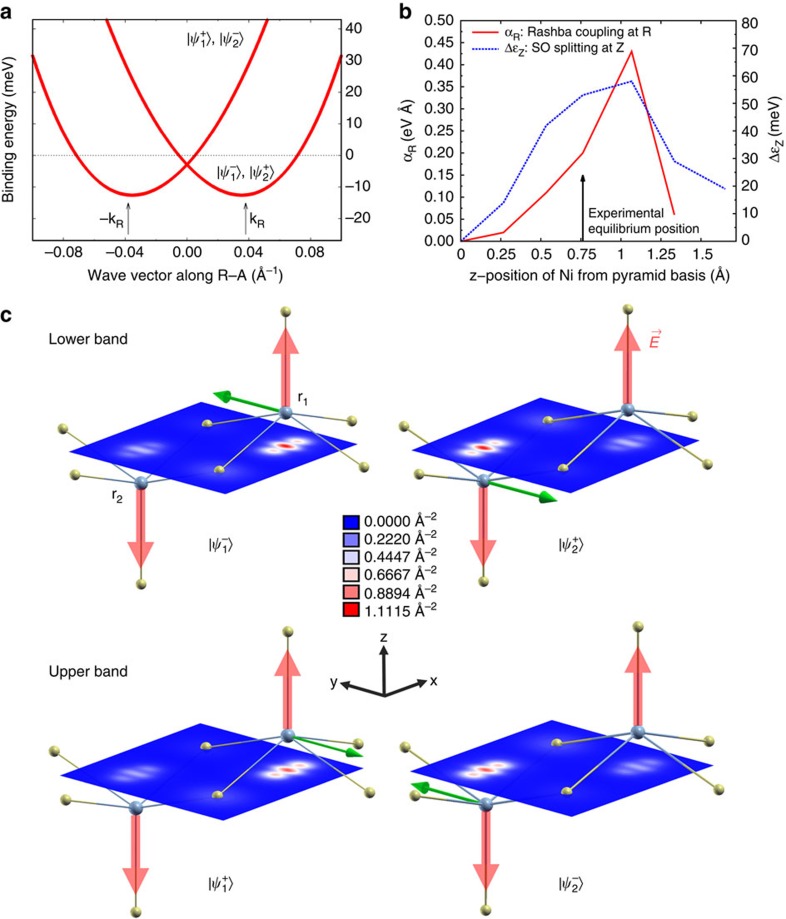
Spinor band structure and Rashba coupling. (**a**) Calculated Rashba-split electron-like A_1_ band at R. The ±*k*_R_ label indicates the *k*-shift of the bottom of the band with respect to R along the R-A direction and *n*=1,2 labels the Ni positions, ***r***_1_ and ***r***_2_, in the unit cell where the spinor 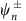 is localized. The dotted line indicates the Fermi level. (**b**) Spin-orbit splitting at Z and Rashba coupling constant *α*_R_ at R calculated as a function of Ni position along the *z* axis. (**c**) Orbital part and spin moment of the Bloch states 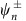 calculated at *k*_R_, where ± denotes the lower and upper energy Rashba branch in **a**. False colours indicate the probability density, 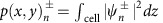, integrated along the *z* axis of the unit cell. Red and green arrows at the two Ni positions **r**_1_ and **r**_2_ indicate the staggered crystal field **E** and the spin moment, respectively. Grey (yellow) balls represent Ni (S) atoms. Ba atoms are not shown for clarity.

Both electron-like and hole-like bands are *k* split along R–A by the same Rashba interaction 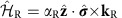, where **k**_R_=**k**–**k**^0^ and **k**^0^ is the wave vector of the R point. While the electron-like upper band would be centred at R in the absence of SO correction, the hole-like band would have a maximum located between R and A. As shown by the present calculations, this difference leads to two different shapes of the spinor band structure, illustrated in Figs 4b and 5a, respectively. In Fig. 5a, the splitting of the electron-like band is decribed by the standard Mexican-hat shape. In Fig. 4b, the calculations predict a displaced split maximum for the hole-like band along the same direction, in agreement with the ARPES data. We fit the latter band using the band dispersion obtained by diagonalising the above Rashba Hamiltonian and find *α*_R_=0.26 eV Å from ARPES, in excellent agreement with the calculated value 0.28 eV Å. The difference between experimental and calculated splitting is due to the limited accuracy of the GGA+*U* approximation in calculating the unperturbed (that is, without SO) band dispersion and its mass renormalization. We computed the Rashba parameters also for the Mexican-hat splitting of the electron-like band, barely visible experimentally at *ɛ*_F_. For this band, we obtained *α*_R_=0.19 eV Å, *k*_R_=0.04 Å^−1^ for the position of the minimum of the band with respect to R, and *ɛ*_R_=11 meV for the depth of the Mexican hat (Fig. 5a).

Contrary to the usual Rashba case, here the spin degeneracy is not lifted, even for the electron-like Mexican-hat band, because every energy level is at least twofold degenerate due to the two-fold multiplicity of the Ni site. It follows that the Rashba Hamiltonian involves two—instead of one—spinors. Our calculations show (Fig. 5c) that the corresponding Bloch states, 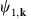 and 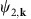, are strongly localized either at ***r***_1_ or at ***r***_2_, where the dipolar crystal field (and the NiS_5_ pyramid) has opposite orientation, ±*E*_*z*_, along the *z* direction. Owing to this localization, the Hamiltonian splits into two independent Rashba Hamiltonians, one for each position, with a staggered distribution of Rashba coupling *α*_R_ forming a Rashba crystal in a square lattice. This situation corresponds to a LIA, as discussed by Zhang *et al*.^11^ who called hidden the resulting compensated spin polarization to distinguish it from that of the usual bulk inversion asymmetry and surface inversion asymmetry cases.

In the present case, the spin chirality at the two Ni positions, ***r***_1_ and ***r***_2_, is opposite, so the net spin polarization of the unit cell is zero. For the Bloch state at ***r***_1_, where *E*_*z*_>0, the solution for the Rashba eigenspinor at a given wave vector **k** reads


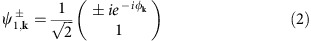


where the superscript ± denotes the upper and lower energy branch, respectively, and 
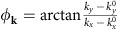
. Since *E*_*z*_ has opposite sign at ***r***_2_, it follows that 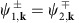; hence, each Rashba branch is made of two opposite spinors whose spatial part is assigned to one of the two Ni positions. This is illustrated in Fig. 5c, where the spatial and spin parts of both Bloch states are plotted in real space at **k**=
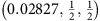
 (crystal units), where the splitting is largest. At this **k** point, *φ*=0, so, according to equation (2), the Rashba spin moment is oriented along the *y* direction with 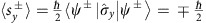. This picture is nicely confirmed by our *ab initio* calculations. The Bloch states projected on a given Ni position yield a local spin moment 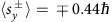 for the two branches, while 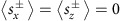. The slight reduction of *s*_*y*_ with respect to the expected 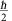 value for the electron is due to the almost complete localization of each Bloch state around one Ni position (Fig. 5c). Finally, one notes that spin-chiral polarized states can be formed by applying a magnetic or electric field.

Using a point-like model for the S anion and for the Ba and Ni cations, the dipolar crystal field at the Ni site is estimated to be *E*_*z*_≈1.4 V Å^−1^. This huge value accounts for the large Rashba effect observed and for the stability of the structure. Indeed, the staggered orientation of the NiS_5_ pyramids minimizes the Coulomb repulsion between apical sulphur atoms. The resulting staggered field configuration turns out to be a very effective LIA mechanism. Thus, the magnitude of the Rashba effect observed reflects the magnitude of the cohesive energy of the crystal. To justify quantitatively this picture, using the GGA–DFT method, we calculated the dependence of the Rashba coupling *α*_R_ at R and the SO splitting Δ*ɛ*_Z_ at Z as a function of the Ni position, *z*, along the *c* axis within the NiS_5_ pyramid. The result reported in Fig. 5b shows that the maximum values for both *α*_R_ and Δ*ɛ*_z_ are obtained close to the equilibrium position of Ni, which means that the most stable structure corresponds to the largest Rashba splitting. This observation suggests an analogy with the Jahn-Teller systems where a gain of electronic energy drives a strain field that splits degenerate electronic states; here, the crystal field is the cause of the splitting.

Following Zhang *et al*.^11^, the present observation of Rashba effect in a centrosymmetric crystal arises from the LIA C_4v_ at the Ni site caused by the above staggered field, which contrasts the usual case of Rashba band splitting at Γ in noncentrosymmetric crystals. The availability of a huge crystal field at the electronically active Ni site extends the possibilities of Rashba band engineering to light elements. The Rashba couplings obtained here could be further enhanced by substituting Ni for heavier 4*d* or 5*d* ions. To verify this expectation, we carried out preliminary GGA–DFT *ab initio* calculations on the hypothetical compounds BaPdS_2_ and BaPtS_2_ assuming that the crystal structure would be similar to that of BaNiS_2_. Our expectation is confirmed by a significant enhancement of the Rashba coupling for the electron-like band; namely, we obtain *α*_R_=0.35 and 0.73 eV Å for the relaxed structure of the Pd and Pt compound, respectively. The corresponding band splittings are also dramatically enhanced up to Δ*ɛ*≈ 0.5 eV in both compounds, which would enable a band structure engineering of solids in view of spintronics applications.

## Methods

### Single-crystal growth

High-purity BaNiS_2_ single crystals were synthesized by a self-flux method, as described elsewhere^27^,^28^. In brief, we first prepared a slightly off-stoichiometry mixture of finely grinded high-purity BaS (99.9%), Ni (99.999%) and S (99.9995%) powders in a graphite crucible. The chosen Ba:Ni:S=0.10:0.425:0.475 composition ensures the crystal growth through the liquidus line of the pseudobinary phase diagram BaNiS_2_-2/3 Ni_3_S_2+δ_ system as shown in ref. 27. The crucible was then sealed under high vacuum at typical pressures *P*∼10^−5^ mbar in a silica tube, heated to 1,000 °C during 2 days and then quenched into water. The product was then grinded and pressed into a pellet for a second time by adding a 10% molar excess of sulphur and the aforementioned heat treatment was repeated. The silica tube was then slowly cooled from 1,000 °C down to 800 °C at a rate of 0.5–1 °C h^−1^ and then quenched into water. The as-prepared melt contains platelet-like shiny black crystals of typical size 1 × 1 × 0.1 mm^3^ that can be mechanically removed from the melt. The crystalline quality, chemical composition and impurity concentration were checked by means of single-crystal X-ray diffraction, energy dispersive X-ray analysis and resistivity measurements in the van der Pauw configuration.

### Angular resolved photoemission spectroscopy

The ARPES experiment was carried out on crystals freshly cleaved *in situ* within the *ab*-plane at 100 K in ultra-high vacuum, at pressures of 10^−11^ mbar or better, at the BaDElPh beamline of the Elettra synchrotron in Trieste, Italy, and at the Cassiopée beamline of the SOLEIL synchrotron in Saint-Aubin, France. In the former experiment, we used an incident photon energy *hv*=26 eV with a Δ*ɛ*=5 meV photon energy resolution. To determine the parity of the bands, the experiment was performed in both *p* and *s* photon polarizations. In the former case, the incoming electric field, perpendicular to the photon momentum, lies within the scattering plane defined by the photon momentum and by the photoelectron momentum. In the latter case, the electric field is perpendicular to the scattering plane. In our geometry, the *p* polarization forms an angle of 45° with the *ab*-plane when at normal emission, while the *s* polarization is parallel to this plane. In the experiment conducted at SOLEIL, the photon energy was 100 eV and the photon energy resolution was Δ*ɛ*=10 meV.

### *Ab initio* calculations

The *ab initio* calculations have been carried out in the GGA–DFT framework with the Perdew–Burke–Ernzerhof functional, implemented in the Quantum ESPRESSO package^29^. In the GGA+*U* calculations, we used the rotationally invariant formalism^30^ with the Hubbard matrix correlating the Ni 3*d* orbitals and fulfilling the atomic spherical symmetries. The Hubbard parameters are *U*=3 eV, *J*=0.95 eV, and the *F*_4_/*F*_2_ Slater integral ratio is taken equal to the atomic value. Ni, Ba and S atoms are described by norm-conserving pseudopotentials. The Ni pseudopotential has 10 valence electrons (4*s*^2^ 3*d*^8^) and non linear core corrections. For Ba, the semi-core states have been explicitely included in the calculations. The S pseudopotential is constructed with the 3*s*^2^ 3*p*^4^ in-valence configuration. In the GGA+*U* calculations with SO coupling, the Ni pseudopotential is fully relativistic and a non-collinear spin (spinor) formalism is used in the DFT framework. The geometry of the cell and the internal coordinates are taken from the experimental values reported previously^14^. The plane-wave cutoff is 120 Ry for the wave function and 500 Ry for the charge. A 8 × 8 × 8 electron-momentum grid and a Methfessel–Paxton smearing of 0.01 Ry are used in the electronic integration during the self-consistent loop. Further calculations with a 16 × 16 × 16 electron-momentum grid and tetrahedra interpolation have been performed for a more accurate determination of the Fermi level starting from a previously converged 8 × 8 × 8 self-consistent electron density. For band structure and Fermi surface calculations, we carried out a Wannier interpolation of the *ab initio* band structure by means of the Wannier90 (ref. 31) program on a *N*_*w*_=4 × 4 × 4 electron-momentum mesh by including all Ni *d* and S *p* states in a 11 eV energy window, which allows to obtain highly accurate interpolated bands.

## Additional information

**How to cite this article:** Santos-Cottin, D. *et al*. Rashba coupling amplification by a staggered crystal field. *Nat. Commun.* 7:11258 doi: 10.1038/ncomms11258 (2016).
